# Factors influencing the use of the “not for generic substitution” mention for prescriptions in primary care: a survey with general practitioners

**DOI:** 10.1186/s12913-018-3652-2

**Published:** 2018-11-12

**Authors:** Virgil Beauvais, Annabelle Marque, Guillaume Ferté, Jan Chrusciel, Julie Souille, Pierre Nazeyrollas, Stéphane Sanchez

**Affiliations:** 1 General Practice, 10000 Troyes, France; 2 Emergency Department, Centre Hospitalier de Troyes, 10000 Troyes, France; 3 Emergency Department, Centre Hospitalier Universitaire de Reims, 51092 Reims, France; 4 Cardiology Department, Centre Hospitalier Universitaire de Reims, 51092 Reims, France; 5 Medical Information Department, Centre Hospitalier de Troyes, 10000 Troyes, France; 6 Troyes, France

**Keywords:** Generic drugs, Prescribing, Not for generic substitution

## Abstract

**Background:**

Generic drug substitution is a public health policy challenge with high economic potential. Generic drugs are generally cheaper than brand-name drugs. Drugs are a significant part of the total health expenditure, especially in ambulatory care. We conducted a cross-sectional study with general practitioners in the Champagne-Ardenne region to determine physician-related factors and beliefs causing doctors to use the Not for Generic Substitution (NGS) mention.

**Methods:**

Questionnaires were sent to General Practitioners (GPs) practicing in Champagne-Ardenne via 3 shipments, from January 2015 to May 2015. Prescriber characteristics and beliefs influencing the use of the NGS mention were assessed for frequent (≥ 5%) and less frequent (< 5%) users of the NGS mention.

**Results:**

Factors associated with above average NGS mention use in bivariate analysis included patient comorbidity, polypharmacy, a concern that generic and brand-name drugs are not bioequivalent and belief in higher efficacy of the brand name drug. The use of an e-prescribing system (EPS) and medical practice in rural areas appeared to be associated with lower use of NGS mention in bivariate analysis but not in multivariable analysis. In multivariable analysis, patient request was associated with a higher use of the NGS mention (NGS ≥ 5%, adjusted Odds Ratio (aOR) = 2.52; 95% CI = [1.46–4.35]; *p* = 0.001), which was also linked to patient age over 65 (NGS ≥ 5%, aOR = 2.33; 95% CI = [1.03–5.30]; *p* = 0.04). The NGS mention was often used for drugs where substitution is debated in the literature (thyroid hormones, antiepileptic drugs).

**Conclusion:**

This work highlights the involvement of the doctor-patient pair for the use of the NGS mention. Patient request was the major reason for using the NGS mention, even though it was not always endorsed by prescribers. Further studies are needed to assess patient views on generic drugs and drug substitution, accounting for their health status and socio-economic condition, to help improve the relevance of the information available to them.

**Electronic supplementary material:**

The online version of this article (10.1186/s12913-018-3652-2) contains supplementary material, which is available to authorized users.

## Background

Generic drug substitution is a public health policy challenge with a high economic potential. Generic drugs are generally cheaper than brand-name drugs. Drugs are a significant part of the total health expenditure, especially in ambulatory care, amounting to € 34.3 billion in France for the year 2012 [1]. The substitution of brand-name drugs is accomplished by the issue of generic drugs, copies of brand-name drugs whose patents are public domain. Generic drugs can be subdivided into: - generic copies (developed by laboratories owning the brand-name patent); − essentially similar generic drugs (differing from the original only by excipients); − assimilated generic drugs (where the form of the active ingredient is changed, for instance esters or isomers of the original active ingredient). All generic drugs must demonstrate bioequivalence to the brand-name molecule through appropriate bioequivalence studies [2]. In France, pharmacists have the right to substitute medications with a generic drug (99–486 decree, June 11, 1999). The generic drug must have the same International Nonproprietary Name (INN), and when possible have the same excipients. Substitutions between generic drugs are also possible, however, a switch between generic drugs may cause greater changes in plasma drug concentrations than generic substitution of reference products [3, 4]. Physicians can exclude the possibility of substitution by affixing the “Not for Generic Substitution” (NGS) mention on prescriptions (French Public Health Code, art. L5125–24 and L5125–23). In France, 4.8–22% of prescriptions contain the NGS mention [5].

Frequent use of the NGS mention burdens the savings gained from the use of generic drugs, with an additional cost of € 100 million in France [6]. The generic market in Europe ranges from 17% in Switzerland to 83% in the United Kingdom [7]. The use of the NGS mention results from the attitude of both participants in the doctor-patient relationship [8]. For prescribers, an opposition to substitution can arise from concerns about the strict bioequivalence between drugs (single-dose studies, studies restricted to healthy volunteers [9]), especially for Narrow Therapeutic Index (NTI) drugs like antiepileptic drugs [10]. Patients may perceive drug substitution as interfering with their usual care. In a survey conducted in Greece in 2015, only 40% of patients agreed that generic drugs are as effective as brand-name drugs [11]. No definite conclusion can be made regarding the effect of a patient’s income on his perception of generic medications [12, 13]. Willingness to be treated with a generic drug decreases when the disease is chronic [14]. Older age has been associated with lower likelihood to endorse generic medications for patients in Germany and in Portugal [15, 16], but not in Japan [17]. Finally, a feeling of therapeutic ineffectiveness due to anxiety surrounding the change of prescription (leading to a Nocebo effect [18, 19]) could influence patients’ attitudes towards generic drugs. Our main objective was to identify factors that influence the use of the NGS mention in prescriptions written by General Practitioners (GPs).

## Methods

### Study design

We conducted an observational cross-sectional study from 29 January to 15 June 2015. We sent questionnaires to General Practitioners (GPs) in the Champagne Ardenne region [see Additional file 1]. The questionnaire was developed specifically for this study. The Champagne-Ardenne region is one of the least densely populated regions in France, with a population of 1.33 million. The age structure of the region is similar to the pooled age structure of the country. The physicians were identified with the French Order of Physicians database (accessed on 12 December 2014, available online). Addresses and telephone numbers were extracted from the yearbook.

### Population

We included all self-employed GPs, not excluding those that were occasionally also employed by public or private organisations. Physicians practicing exclusively in hospitals were excluded. According to the Order of Physicians atlas of medical demographics of the Champagne-Ardennes region, there were 1216 general practitioners in the region [20]. GPs who specialized in other fields (e.g. vascular medicine) or who practiced alternative/complementary medicines (traditional Chinese medicine, homeopathy ...) were excluded. Our first mailing included 801 (79.6%) postal mails and 205 (20.4%) e-mails. We sent two reminders for non-responders in March and April 2015.

### Primary endpoint and collected variables

Our primary endpoint was the average rate of prescriptions containing the NGS mention (number of prescription with the NGS mention at least once / total number of prescriptions). Participants reported their frequency of use of the NGS mention by answering a multiple-choice question in the survey (the answers were coded < 5%; 5–15%; 15–25%; > 25%). The results of the survey were based on the answers provided by physicians and not on a study of individual prescriptions. We identified two groups of physicians based on their declared use of the NGS mention, the threshold value being the average national rate (5%). Physicians who reported a frequency of NGS mention ≥5% were considered frequent users of the NGS mention. The rationale behind NGS mention use was provided by physicians by answering the question: “Which situations led you to use the NGS mention?”. For each situation justifying the use of the NGS mention in the list, the physician indicated how frequently it was a reason for not substituting medication. The reasons for not substituting expressed by prescribers were classified as follows:

- Higher efficacy of the brand-name drug compared to the generic drug (prescriber’s assessment)
- Concern that the generic drug is not bioequivalent to the brand-name drug
- Occurrence of benign adverse effects (AEs) observed by the prescriber
- Occurrence of serious AEs (overdose, anaphylactic reaction, blood abnormalities...) observed by the prescriber
- Scientific guidelines advising against substitution (peer-reviewed articles, regulatory agency guidelines …)
- Patient’s request: when the patient objects to substituting the drug
- Existence of a risk when switching related to:
  - ○ Patient comorbidity
  - ○ Polypharmacy and risk of drug interactions
  - ○ Age of the patient over 65.
- Prescriber’s personal choice for other reasons (routine, desire to use drug names the patient is familiar with …).

Physicians selected their answer in a list containing the following: never (0%); rarely (1–24%), sometimes (25–49%); often (50–74%); very often / always (75–100%). Prescriber-related variables that were collected included:

- Age and sex of prescribers. Age was treated as a continuous variable.
- Rate of INN prescriptions: lower or higher than 15% of total prescriptions (national average in 2013: 13.2%)
- Participation in a quality circle with reviews of clinical cases
- The use of E-Prescribing system software (EPS)
- Setting (city/ rural setting).

Physicians were also asked to select in a multiple-choice question the most frequent reason for using the NGS mention for 18 distinct therapeutic classes (antiplatelet agents, thyroid hormones, antiepileptic drugs, antiacids, antispasmodics, step I analgesics, step II and III analgesics, NSAIDs / SAIDs, antibiotics, antidepressants, hypnotics, antipsychotics, lipid-lowering agents, oral anti-diabetic drugs, diuretics, Angiotensin Converting Enzyme inhibitors or Angiotensin II Receptor Blockers, beta blockers, oral contraceptives).

In the descriptive analysis, continuous variables were reported with their mean and standard deviation; categorical variables were presented as absolute frequencies and percentages. Qualitative variables were analyzed by the Chi-squared test or Fisher’s exact test as appropriate. Student’s *t* test or the Wilcoxon-Mann-Whitney rank-sum test was used to analyze the relationship between continuous variables. A multivariable analysis using logistic regression was performed to test the relationship between an NGS prescription rate ≥ 5% and the following variables: Practice in cities (versus practice in rural areas); the use of e-prescribing software; INN < 15%; higher efficacy of brand-name drug; non-bioequivalence with brand-name drug; patient’s request; patient comorbidities; polypharmacy and patient’s age over 65. Variables included in the models were significant in univariate analysis with *p* <  0.10. Missing data was treated by listwise deletion. The analysis was performed with SPSS v20.0 (IBM Corp, Armonk, NY).

## Results

### Population

We obtained 277 valid responses. The response rate was 27.5%. Five of the surveyed physicians no longer practiced in the region. 64.3% of physicians in our study were male, 35.7% were female (Table 1). Mean physician age was 52.2 years (SD 11.1). 53.8% worked in rural areas versus 46.2% in cities. Group practice was the most frequent type of practice (66.8% vs 33.2%).

**Table 1:** Characteristics of the surveyed physicians

Characteristic	n or mean (total *n* = 277)	% or SD
Sex: Female – n, %	99	(35.7)
Age: mean (SD)	52.2	(11.1)
Setting: City (versus rural area) – n, %	128	(46.2)
Type of practice: Alone (versus group practice) – n, %	92	(33.2)
Use of Electronic Prescription Software (EPS): Yes – n, %	235	(84.8)
Use of INN^a^: ≥ 15%		
– n, %	156	(56.3)
Use of the NGS^b^ mention		
– n, %	–	–
< 5%	119	(43.0)
≥ 5%	158	(57.0)
5–15%	102	(36.8)
15–25%	33	(11.9)
> 25%	23	(8.3)

^a^*INN* International Nonproprietary Name

^b^*NGS* “Not for Generic Substitution”

#### Factors related to prescribers

84.8% of prescribers used an EPS. The use of an EPS was more frequent in physicians who used NGS in less than 5% of prescriptions (91.6% vs 79.8%) (Table 2).39.5% of low NGS mention users practiced in a city (60.5% in a rural setting), while 51.9% of high NGS mention users practiced in a city (48.1% in a rural setting). Practicing in a city was significantly associated with a higher NGS mention use (*p* = 0.04). Age, gender and participation in a quality circle did not seem to influence the use of the NGS mention in our study.

**Table 2:** Bivariate analysis of factors related to prescribers according to the NGS rate

Variable	NGS^a^ mention < 5% of prescriptions *n* = 119	NGS^a^ mention ≥5% of prescriptions *n* = 158	*p* value
Age of prescriber (mean, SD^b^)	53.2 (10.8)	51.5 (11.3)	0.44
Sex: Female (n, %)	36 (30.3)	63 (39.9)	0.10
Use of INN^c^: < 15% (n, %)	44 (37.0)	77 (48.7)	0.05
Participation in a quality circle: Yes (n, %)	77 (64.7)	87 (55.1)	0.11
Use of EPS^d^: Yes (n, %)	109 (91.6)	126 (79.8)	0.006
Place of practice: City (versus rural settings) (n, %)	47 (39.5)	82 (51.9)	0.04

^a^*GS* Not for Generic Substitution

^b^*SD* Standard Deviation

^c^*INN* International Nonproprietary Name

^d^*PS* E-Prescription System

### General reasons for not substituting expressed by prescribers

Table 3 shows the reasons for not substituting expressed by physicians according to their rate of NGS mention use. Using the NGS mention for fear of benign adverse effects (*p* = 0.22) or severe adverse effects (*p* = 0.59), or because of regulatory agencies best practice recommendations (*p* = 0.21), or following the physician’s personal choice for other reasons (routine, desire to use drug names the patient is familiar with …, *p* = 0.12), were not predictive of the NGS mention rate in prescriptions. The fear of non-equivalence between generic drugs and brand-name drugs, and belief in a higher efficacy of the brand-name drug were predictive of NGS mention use (*p* = 0.02 and *p* = 0.03). Patient’s request for the brand name-drug was frequently given as a reason for using the NGS mention, especially among prescribers who often used the NGS mention (65.0% vs 39.8%; *p* <  0.001). The patient’s condition also influenced prescribers: age over 65, comorbidity and polypharmacy were legitimate reasons for using the NGS mention for physicians that often used the expression (*p* <  0.001). The data is available [see Additional file 2].

**Table 3:** Reasons for not substituting indicated by frequent (≥ 5%) and less frequent (< 5%) users of the Not for Generic Substitution mention

Reasons for not substituting medication ^a^	NGS ^b^ mention < 5%: n (%)	NGS ^b^ mention ≥5%: n (%)	*p* value
Patient age over 65	17 (14.5)	69 (44.0)	< 0.001
Higher efficacy of the brand-name drug	19 (16.1)	44 (28.2)	0.02
Does not believe in bioequivalence	21 (17.7)	45 (28.9)	0.03
Benign adverse effect	21 (17.7)	37 (23.7)	0.22
Severe adverse effect	27 (22.7)	31 (20.0)	0.59
Monographies and scientific journals	7 (6.2)	15 (10.1)	0.26
Regulatory agency guidelines	11 (9.6)	8 (5.5)	0.21
Advice by specialist	20 (17.2)	32 (20.7)	0.48
Patient request	47 (39.8)	102 (65.0)	< 0.001
Patient comorbidity	11 (9.3)	56 (36.4)	< 0.001
Polypharmacy	16 (13.7)	61 (39.4)	< 0.001
Physician’s personal choice for other reasons (routine, desire to use drug names the patient is familiar with …)	4 (3.4)	12 (7.9)	0.12

^a^Very often/always a reason for not substituting medication. Percentages were calculated using available data

^b^*NGS* Not for Generic Substitution

#### Multivariable analysis

After adjusting for confounders, patient request (OR 2.52; 95% CI 1.46–4.35; *p* = 0.001), and patient age (OR = 2.33; 95% CI 1.03–5.30; *p* = 0.04) were significantly associated with a greater use of the NGS mention (Table 4). Similar trends were noted for patient comorbidity (OR = 2.79; 95% CI 0.96–8.04; *p* = 0.06) and practice in urban areas (OR 1.59; 95% CI 0.93–2.74; *p* = 0.09). Although this result did not reach statistical significance, the use of an EPS for prescriptions also seemed to be associated with a lower use of the NGS mention (< 5%), (OR = 0.46; 95% CI 0.20–1.05; *p* = 0.07).

**Table 4:** Multivariable analysis of factors associated with NGS ≥ 5%

Variable	Adjusted Odds Ratio (aOR)	95% Confidence Interval (95% CI)	*p* value
INN^a^: < 15%	1.08	0.62–1.90	0.78
Practice: in cities (versus rural areas)	1.59	0.93–2.74	0.09
EPS^b^	0.46	0.20–1.05	0.07
Higher efficacy of brand-name drug	1.52	0.70–3.30	0.29
Does not believe in bioequivalence	0.92	0.41–2.05	0.84
Patient request	2.52	1.46–4.35	0.001
Patient comorbidity	2.79	0.96–8.04	0.06
Polypharmacy	1.00	0.37–2.73	0.99
Age of patient over 65 years	2.33	1.03–5.30	0.04

^a^*INN* International Nonproprietary Name

^b^*EPS* e-Prescription System

### Differences between therapeutic classes

The average rate of using the NGS mention very often/always for the 18 classes in our study was 5.8% (Table 5). 48.4% of prescribers in our study reported using the NGS mention very often/always when prescribing thyroid hormones, 28.5% when prescribing antiepileptic drugs, 7.9% when prescribing antiplatelet agents. Classes for which physicians and/or pharmacists perceived additional revenues when substituted (ROSP: antidepressants, antibiotics, antiacids, antihypertensive medication, statins) were not different from other classes regarding frequency of NGS use.

**Table 5:** Differences between therapeutic classes regarding the frequency of the “Not for Generic Substitution” mention use

Therapeutic class	NGS mention use				
	Never (0%)	Rarely (1–24%)	Sometimes (25–49%)	Often (50–74%)	Very often/always (75–100%)
Thyroid hormones – n, %	21 (7.6)	30 (10.8)	30 (10.8)	62 (22.4)	134 (48.4)
Antiepileptic drugs – n, %	31 (11.2)	51 (18.4)	48 (17.3)	68 (24.6)	79 (28.5)
Antiplatelet agents – n, %	45 (16.2)	110 (39.7)	40 (14.4)	60 (21.7)	22 (7.9)
Proton pump inhibitors – n, %	74 (26.7)	117 (42.2)	40 (14.4)	38 (13.7)	8 (2.9)
Neuroleptics – n, %	95 (34.3)	107 (38.6)	44 (15.9)	25 (9.0)	6 (2.2)
Oral antidiabetic drugs– n, %	85 (30.7)	115 (41.5)	48 (17.3)	23 (8.3)	6 (2.2)
Statins – n, %	88 (31.8)	124 (44.8)	38 (13.7)	22 (7.9)	5 (1.8)
Antidepressants – n, %	74 (26.7)	117 (42.2)	57 (20.6)	25 (9.0)	4 (1.4)
Hypnotic drugs – n, %	80 (28.9)	125 (45.1)	40 (14.4)	29 (10.5)	3 (1.1)
Angiotensin converting enzyme inhibitors/Angiotensin II receptor blockers – n, %	81 (29.2)	120 (43.3)	48 (17.3)	25 (9.0)	3 (1.1)
Step II-III analgesics – n, %	106 (38.3)	109 (39.4)	47 (17.0)	12 (4.3)	3 (1.1)
Diuretics – n, %	106 (38.3)	123 (44.4)	31 (11.2)	15 (5.4)	2 (0.7)
Antispasmodic drugs – n, %	139 (50.2)	96 (34.7)	30 (10.8)	10 (3.6)	2 (0.7)
Anti-inflammatory drugs (Nonsteroidal and steroidal) – n, %	118 (42.6)	108 (39.0)	40 (14.4)	9 (3.2)	2 (0.7)
Beta blockers – n, %	88 (31.8)	128 (46.2)	36 (13.0)	24 (8.7)	1 (0.4)
Antibiotics – n, %	107 (38.6)	108 (39.0)	39 (14.1)	22 (7.9)	1 (0.4)
Step I analgesics – n, %	138 (49.8)	100 (36.1)	25 (9.0)	13 (4.7)	1 (0.4)
Oral contraceptives – n, %	116 (41.9)	114 (41.2)	34 (12.3)	13 (4.7)	0 (0.0)

Biological or clinical inequivalence to the brand-name drug was the main reason stated for not substituting thyroid hormones and a frequent reason for not substituting antiepileptic drugs (22.4%) (Table 6). For antiplatelet agents, the main reason was compliance with specialist prescription (34.5%).

**Table 6:** Most frequent reasons for using the NGS mention for 18 therapeutic classes

Drug	Most frequent reasons for using the NGS^a^ mention					
	Patient request	Benign adverse drug reaction	Severe adverse drug reaction	Biological or clinical inequivalence to the brand-name drug	Class with increased risk (inter-individual variations, interactions, narrow therapeutic range …)	Compliance with specialist prescription
Antiplatelet agents - n, %	64(27.6)	11 (4.7)	8 (3.4)	28 (12.1)	41 (17.7)	80 (34.5)
Thyroid hormones - n, %	29 (11.3)	7 (2.7)	3 (1.2)	105 (41.0)	73 (28.5)	39 (15.2)
Antiepileptic drugs - n, %	41 (16.7)	2 (0.8)	3 (1.2)	55 (22.4)	91 (37.0)	54 (22.0)
Antiacids - n, %	112 (55.2)	31 (15.3)	3 (1.5)	47 (23.2)	6 (3.0)	4 (2.0)
Antispasmodic drugs - n, %	106 (76.8)	14 (10.1)	1 (0.7)	14 (10.1)	2 (1.4)	1 (0.7)
Step I analgesics - n, %	107 (77.0)	14 (10.1)	1 (0.7)	9 (6.5)	6 (4.3)	2 (1.4)
Step II/III analgesics - n, %	104 (60.8)	30 (17.5)	3 (1.8)	24 (14.0)	7 (4.1)	3 (1.8)
Anti-inflammatory drugs - n, %	89 (56.0)	34 (21.4)	8 (5.0)	17 (10.7)	10 (6.3)	1 (0.6)
Antibiotics - n, %	86 (50.3)	45 (26.3)	6 (3.5)	24 (14.0)	8 (4.7)	1 (0.6)
Antidepressants - n, %	110 (54.2)	28 (13.8)	2 (1.0)	33 (16.3)	17 (8.4)	13 (6.4)
Hypnotics/Benzodiazepines - n, %	143 (72.6)	15 (7.6)	1 (0.5)	23 (11.7)	10 (5.1)	5 (2.5)
Neuroleptics - n, %	94 (51.6)	20 (11.0)	2 (1.1)	18 (9.9)	26 (14.3)	22 (12.1)
Statins - n, %	105 (55.6)	42 (22.2)	8 (4.2)	16 (8.5)	10 (5.3)	8 (4.2)
Oral anti-diabetic drugs - n, %	83 (43.2)	55 (28.6)	2 (1.0)	30 (15.6)	14 (7.3)	8 (4.2)
Diuretics - n, %	102 (59.6)	22 (12.9)	2 (1.2)	20 (11.7)	14 (8.2)	11 (6.4)
Beta blockers - n, %	94 (49.7)	25 (13.2)	3 (1.6)	29 (15.3)	19 (10.1)	19 (10.1)
Angiotensin-converting-enzyme inhibitors/ Angiotensin II receptor blockers - n, %	100 (51.0)	35 (17.9)	1 (0.5)	25 (12.8)	13 (6.6)	22 (11.2)
Oral contraceptives - n, %	110 (68.3)	19 (11.8)	2 (1.2)	16 (9.9)	12 (7.5)	2 (1.2)

^a^*NGS* Not for Generic Substitution

### Physicians’ opinion on generic drugs and patients’ requests for not substituting medications

Regarding concerns about the equivalence of generic drugs with their brand-name counterpart, 39.7% of physicians were worried about differences in bioavailability whereas 10.8% were preoccupied by inter/intra-individual pharmacokinetic variations. Patients’ requests for the NGS mention were seen as fully justified by 2.5% of physicians who rarely used the NGS mention, versus 14.0% of physicians who often used the NGS mention (*p* <  0.001). Therefore, it appears that physicians might have used the NGS mention even though they were not entirely convinced it was necessary. 75.1% of physicians in our study reported they would try to convince their patients to accept generic substitution when it was possible to do so. The proportion was greater among less frequent users of the NGS mention (NGS < 5%: 81.5%; NGS ≥ 5%: 70.3%; *p* <  0.05). Finally, when patients asked for the NGS mention, 57.7% of physicians believed they could convince them to change their mind. 58.5% of physicians feared a deterioration of their relationship with the patient if they refused to comply with the request.

## Discussion

Our study shows that the patient’s opposition to the substitution is not only a very frequent pattern of recourse to NGS mention, but also the most significant factor associated with higher use of the NGS term among prescribers. The use of the NGS mention therefore results from a negotiation between the patient and the physician. Patients’ distrust of generic drugs has been described in qualitative and quantitative studies. Some patients see them as counterfeit medicines, less powerful than brand-name drugs [21]. Their number and changing names make it difficult for patients to remember them, and patients are preoccupied by their composition and origin [21]. In a study by Iskounen et al. [22], 27% of patients thought that generic drugs were less effective than brand-name drugs, and 15.8%held a negative view of generic drugs. In June 2018, a patient association alerted regulatory agencies that the new formula of a drug marketed in France caused adverse effects and/or therapeutic imbalance [23]. The active principle of the drug and dose were theoretically the same as the ancient formula. Patients had the option to use generic forms of the drug instead, however they were not convinced of their efficacy. This underlines the importance of identifying the factors that influence patients’ opinions about generic drugs.

A study conducted in Greece shows that patients’ main sources of information about generic drugs are the media, followed by the internet and relatives [11], with health professionals playing a lesser role.

In our study, older patients elicited a greater use of the NGS mention. Patient comorbidity could play a role in the decision not to substitute, although it did not reach statistical significance in our study. Older age and comorbidity may be considered by prescribers and patients alike as leading to a higher risk of therapeutic imbalance. Prescribers practicing in cities tended to use the NGS mention more frequently than those working in rural environments. Because physician density is lower in these areas, prescribers may also feel less pressure to comply with patient requests regarding use of the NGS mention. Another possible reason is that drug sales representatives are less likely to work in rural areas [24]. Conversely, the use of an EPS seemed to entail a lower use of the NGS mention. This may be due to an easier use of INN permitted by EPS for physicians less familiar with the INN [25]. However, many long-term prescriptions are initiated at the hospital where, in France, INN use is very low [26, 27]. Participation in a quality circle (where colleagues can discuss treatment options and review clinical cases) was not associated with a lower use of the NGS mention in our study. In this regard, our findings differ from Spiegel et al. [28], although the length of participation was not measured in our study. Physicians often considered that generic medications were not equivalent to brand-name drugs because of differences in bioavailability (for most drugs the rate and extent of absorption are expected to fall within 80–125% of the brand name drugs’ [29]). Inter/intra-individual pharmacokinetic variations [9] were less frequently cited by physicians in our study.

### Strengths and limitations of this study

This study highlights the factors and beliefs that influence the use of the NGS mention. Our sample was representative of the GPs of Champagne Ardenne in 2015 with respect to age (mean age 51.9 years) and sex (35.5% of women) [20, 30]. We could not appreciate other prescriber characteristics such as the volume of activity or their case mix. Our study included GPs in the Champagne-Ardenne region, where the rate of NGS mention use was 3.2% in 2012, slightly below the national average (4.8%) [5]. Nevertheless, wide regional disparities (8.3% for Basse-Normandie, 1.4% for Pays-De-Loire) may limit the extrapolation of our results to a national level. Non-response was a major limitation of this study. Physicians who were convinced of the quality and safety of generic drugs could have been more likely to complete the survey. This could have increased the reported proportion of NGS mentions motivated by patients’ requests. The authors could not examine the physician’s individual prescriptions, therefore the measures reported in this article only reflect the surveyed physicians’ perception of their prescribing behavior. There could be discrepancies between the real proportion of NGS mentions on prescriptions and the physicians’ estimates.

## Conclusion

Patient request was the most frequent reason expressed by prescribers for using the NGS mention. The difficulty in distinguishing an adverse effect from a nocebo effect, and the risk of a deterioration of the doctor-patient relationship in case of refusal could lead otherwise reluctant prescribers to use the NGS mention. Further studies are needed to assess patient views on generic drugs and drug substitution, accounting for their health status and socio-economic position, to improve the relevance of the information available to them.

## Additional files

Additional file 1: Factors influencing the use of the “Not for Generic Substitution” mention for prescriptions in primary care: questionnaire. English version of the questionnaire used for the survey. (DOCX 34 kb)

Additional file 2: Data from “Factors influencing the use of the “Not for Generic Substitution” term for prescriptions in primary care: a survey with general practitioners”**.** The data from the survey was anonymized. (XLSX 457 kb)

## Abbreviations

ANSM: French regulatory agency for medicines
aOR: Adjusted Odds Ratio
EPS: E-Prescribing System
GPs: General practitioners
INN: International Nonproprietary Name
NGS: Not for generic substitution
ROSP: Payment system based on public health objectives
