# Supplementary material for: Factors influencing the use of the “not for generic substitution” mention for prescriptions in primary care: a survey with general practitioners
Source: BMC Health Serv Res. 2018 Nov 12;18:850. doi: 10.1186/s12913-018-3652-2 (PMC6233580; doi:10.1186/s12913-018-3652-2)
Supplement: Supplementary file 1 — Factors influencing the use of the “Not for Generic Substitution” mention for prescriptions in primary care: questionnaire. English version of the questionnaire used for the survey. (DOCX 34 kb) [file 12913_2018_3652_MOESM1_ESM.docx]

**FACTORS INFLUENCING THE USE OF THE “NOT FOR GENERIC SUBSTITUTION” MENTION FOR PRESCRIPTIONS IN PRIMARY CARE: QUESTIONNAIRE**

**1/ Personal information**

**1.1/ Age**: ___________

**1.2/ Sex**:

🞐 Male

🞐 Female

**1.3/ When did you start working as a general practitioner (year)**: __________ _

**1.4/ How would you describe the location of your practice**:

🞐 urban

🞐 semi urban

🞐 rural

**1.5/ Type of practice**

🞐 I practice alone

🞐 group practice

**1.6/ Continuing Professional Development (CPD)** *(answers are not mutually exclusive)*:

🞐 General Practice scientific publications

🞐 Specialized Medicine scientific publications

🞐 Continuous medical education / Professional development groups with patient chart reviews (at least 1 session/month)

🞐 Other (please clarify): __________________________________ _

🞐 None

**1.7/ Do you use any e-prescribing software?**

🞐 Yes

🞐 No

**2/ Prescriptions**

**2.1/ When you prescribe, do you use the International Non-proprietary Name (INN)?**

🞐 Yes

🞐 No *(go to question* **2.4***)*

**2.2 / If the answer to the previous question was “Yes”, what is the proportion of prescriptions in which you use the INN?** *(1 answer)*

🞐 < 5%

🞐 [5-15%]

🞐 [15-25%]

🞐 > 25%

**2.3 / If you answered “Yes”, what is the main context in which you would use the INN?** *(1 answer)*

🞐 Initiation of treatment

🞐 Renewal of treatment

**2.4 / What is the proportion of prescriptions in which you use the “Not for Generic Substitution” mention for at least one of the drugs prescribed?** *(1 answer)*

🞐 < 5%

🞐 [5-15%]

🞐 [15-25%]

🞐 > 25%

**2.5/ General reasons for not substituting medication: in which situations do you use the “Not for Generic Substitution” mention?** *(1 answer per item)*

|  | NEVER [0%] | RARELY [1-24%] | SOME-TIMES  [25-49%] | OFTEN [50-74%] | ALWAYS  [75-100%] |
| --- | --- | --- | --- | --- | --- |
| - Your own personal choice (*e.g. routine, willingness to use drug names the patient is familiar with ...*) | 🞐 | 🞐 | 🞐 | 🞐 | 🞐 |
| - Higher efficacy of brand-name drug | 🞐 | 🞐 | 🞐 | 🞐 | 🞐 |
| - Brand-name drug is not bioequivalent to generic drug | 🞐 | 🞐 | 🞐 | 🞐 | 🞐 |
| - Benign adverse drug reaction (*e.g. digestive problems, vertigo …*) | 🞐 | 🞐 | 🞐 | 🞐 | 🞐 |
| - Severe adverse drug reaction (*e.g. allergy, drug poisoning*) | 🞐 | 🞐 | 🞐 | 🞐 | 🞐 |
| - Scientific guidelines |  |  |  |  |  |
| - Drug Monographs/specialized publications | 🞐 | 🞐 | 🞐 | 🞐 | 🞐 |
| - Regulatory Agency guidelines | 🞐 | 🞐 | 🞐 | 🞐 | 🞐 |
| - Specialist advice | 🞐 | 🞐 | 🞐 | 🞐 | 🞐 |
| - Patient request | 🞐 | 🞐 | 🞐 | 🞐 | 🞐 |
| - At risk population |  |  |  |  |  |
| - Severe comorbidity | 🞐 | 🞐 | 🞐 | 🞐 | 🞐 |
| - Polypharmacy/drug interactions | 🞐 | 🞐 | 🞐 | 🞐 | 🞐 |
| - Age > 65 years | 🞐 | 🞐 | 🞐 | 🞐 | 🞐 |

**3/ Views on generic drugs**

**3.1/ Frequency of the “Not For Generic Substitution” mention use** *(1 answer per therapeutic class)*

|  | NEVER [0%] | RARELY  [1-24%] | SOME-TIMES  [25-49%] | OFTEN  [50-74%] | ALWAYS  [75-100%] |
| --- | --- | --- | --- | --- | --- |
| - Antiplatelet agents (*e.g. Clopidogrel*) | 🞐 | 🞐 | 🞐 | 🞐 | 🞐 |
| - Thyroid hormones | 🞐 | 🞐 | 🞐 | 🞐 | 🞐 |
| - Antiepileptic drugs | 🞐 | 🞐 | 🞐 | 🞐 | 🞐 |
| - Antiacids | 🞐 | 🞐 | 🞐 | 🞐 | 🞐 |
| - Antispasmodics | 🞐 | 🞐 | 🞐 | 🞐 | 🞐 |
| - Step I analgesics | 🞐 | 🞐 | 🞐 | 🞐 | 🞐 |
| - Step II / III analgesics | 🞐 | 🞐 | 🞐 | 🞐 | 🞐 |
| - NSAIDs / Steroid anti-inflammatory drugs | 🞐 | 🞐 | 🞐 | 🞐 | 🞐 |
| - Antibiotics | 🞐 | 🞐 | 🞐 | 🞐 | 🞐 |
| - Antidepressants | 🞐 | 🞐 | 🞐 | 🞐 | 🞐 |
| - Hypnotics, benzodiazepines | 🞐 | 🞐 | 🞐 | 🞐 | 🞐 |
| - Antipsychotics | 🞐 | 🞐 | 🞐 | 🞐 | 🞐 |
| - Lipid lowering drugs / Statins | 🞐 | 🞐 | 🞐 | 🞐 | 🞐 |
| - Oral anti-diabetic drugs | 🞐 | 🞐 | 🞐 | 🞐 | 🞐 |
| - Antihypertensive drugs |  |  |  |  |  |
| - Diuretics | 🞐 | 🞐 | 🞐 | 🞐 | 🞐 |
| - Beta blockers | 🞐 | 🞐 | 🞐 | 🞐 | 🞐 |
| - Angiotensin Converting Enzyme Inhibitors / Angiotensin II Receptor Blockers | 🞐 | 🞐 | 🞐 | 🞐 | 🞐 |
| - Oral contraceptives | 🞐 | 🞐 | 🞐 | 🞐 | 🞐 |

**3.2/ Please indicate the most frequent reason for using the “Not for Generic Substitution” mention for each medication** *(1 answer per item)*

|  | Patient request | Benign adverse drug reaction | Severe adverse drug reaction | Clinical or biological efficacy not equivalent | At risk drug class (Individual variations, drug interactions, narrow therapeutic index…) | Compliance with specialist prescription |
| --- | --- | --- | --- | --- | --- | --- |
| - Antiplatelet agents *(e.g. Clopidogrel)* | 🞐 | 🞐 | 🞐 | 🞐 | 🞐 | 🞐 |
| - Thyroid hormones | 🞐 | 🞐 | 🞐 | 🞐 | 🞐 | 🞐 |
| - Antiepileptic drugs | 🞐 | 🞐 | 🞐 | 🞐 | 🞐 | 🞐 |
| - Antiacids | 🞐 | 🞐 | 🞐 | 🞐 | 🞐 | 🞐 |
| - Antispasmodics | 🞐 | 🞐 | 🞐 | 🞐 | 🞐 | 🞐 |
| - Step I analgesics | 🞐 | 🞐 | 🞐 | 🞐 | 🞐 | 🞐 |
| - Step II / III analgesics | 🞐 | 🞐 | 🞐 | 🞐 | 🞐 | 🞐 |
| - NSAIDs / Steroid anti-inflammatory drugs | 🞐 | 🞐 | 🞐 | 🞐 | 🞐 | 🞐 |
| - Antibiotics | 🞐 | 🞐 | 🞐 | 🞐 | 🞐 | 🞐 |
| - Antidepressants | 🞐 | 🞐 | 🞐 | 🞐 | 🞐 | 🞐 |
| - Hypnotics, benzodiazepines | 🞐 | 🞐 | 🞐 | 🞐 | 🞐 | 🞐 |
| - Antipsychotics | 🞐 | 🞐 | 🞐 | 🞐 | 🞐 | 🞐 |
| - Lipid lowering drugs / Statins | 🞐 | 🞐 | 🞐 | 🞐 | 🞐 | 🞐 |
| - Oral anti-diabetic drugs | 🞐 | 🞐 | 🞐 | 🞐 | 🞐 | 🞐 |
| - Antihypertensive drugs |  |  |  |  |  |  |
| - Diuretics | 🞐 | 🞐 | 🞐 | 🞐 | 🞐 | 🞐 |
| - Beta blockers | 🞐 | 🞐 | 🞐 | 🞐 | 🞐 | 🞐 |
| - Angiotensin Converting Enzyme Inhibitors / Angiotensin II Receptor Blockers | 🞐 | 🞐 | 🞐 | 🞐 | 🞐 | 🞐 |
| - Oral contraceptives | 🞐 | 🞐 | 🞐 | 🞐 | 🞐 | 🞐 |

**3.3/ In your opinion, which of the factors listed below could explain a difference in biological or clinical efficacy between generic drugs and brand-name drugs** *(1 answer)*.

🞐 Effect of excipients

🞐 Higher variation of active principle concentration because of bioequivalence norms

🞐 Difference in manufacturing of generic drugs

🞐 Individual variations

🞐 Unknown drug interactions

**4/ Development of generic drug prescription**

**Which factors could increase the proportion of generic drugs in your prescriptions?** *(1 answer per item*)

|  | **Yes** | **No** |
| --- | --- | --- |
| - Publication of bioequivalence studies (internet, scientific publications) | 🞐 | 🞐 |
| - Exclusion of “at risk” medications from automatic substitution (narrow therapeutic index, high interindividual variability) | 🞐 | 🞐 |
| - More clinical studies (efficacy, tolerance) | 🞐 | 🞐 |
| - Financial incentives | 🞐 | 🞐 |
| - Informing patients about generic drugs | 🞐 | 🞐 |
| - All prescriptions should be written using the International Non-proprietary Name (INN) (to ensure patients are used to the INN) | 🞐 | 🞐 |
| - Standardizing the pharmaceutical formulation of medication | 🞐 | 🞐 |

**5/ Study of the patient-doctor relationship when the patient requests a brand-name drug**

**5.1/ How old are patients who refuse generic substitution?** *(please answer with a number from 1 to 5: 1 = more frequent, 5 = less frequent)*

- > 65 - from 1 to 5:

- 40-65 - from 1 to 5:

- 25-40 - from 1 to 5:

- < 25 - from 1 to 5:

- Children - from 1 to 5:

**5.2/ Does the patient’s request seem justified to you?** *(1 answer)*

🞐 NEVER [0%]

🞐 RARELY [1-24%]

🞐 SOMETIMES [25-49%]

🞐 OFTEN [50-74%]

🞐 ALWAYS [75-100%]

**5.3/ Is the patient’s request retroactive? (the patients who refuse generic medication come back for a new prescription with the “Not for Generic Substitution” mention after seeing the pharmacist)** *(1 answer)*

🞐 NEVER [0%]

🞐 RARELY [1-24%]

🞐 SOMETIMES [25-49%]

🞐 OFTEN [50-74%]

🞐 ALWAYS [75-100%]

**5.4/ If you think substitution is possible, do you try to convince the patient that the generic drug is equivalent to the brand-name drug?** *(1 answer)*

🞐 Yes *(go to question* **5.5***)*

🞐 No *(go to question* **5.6***)*

**5.5/ If you answered yes, do you manage to convince the patient that he/she does not need the “Not for Generic Substitution” mention on his/her prescription?** *(1 answer)*

🞐 Yes

🞐 No

**5.6/ If you answered no, please explain why?** *(1 answer)*

🞐 Fear of deteriorating the relationship with the patient

🞐 Lack of time

🞐 Older patient is used to the brand-name drug

🞐 Retroactive patient request between scheduled consultations

🞐 Patients have a right to choose the brand-name drug if they wish to do so

**5.7/ Do you think opposite views regarding the “Not for Generic Substitution” mention can adversely affect your relationship with the patient?** *(1 answer)*

🞐 Yes

🞐 No

**5.8/ When the patient requests the “Not for Generic Substitution” mention, what is the reason?**

*(answer with a number from 1 to 4 : 1 = more frequent, 4 = less frequent)*

- Adverse drug reactions - from 1 to 4:

- Lower clinical efficacy - from 1 to 4:

- A negative view of the product’s quality - from 1 to 4:

- Practical difficulties regarding drug formulation or drug name - from 1 to 4:

**5.9/ Are patients well informed regarding generic drugs?** *(1 answer)*

🞐 Yes

🞐 No

Thank you for your participation
